# Mastering algebra retrains the visual system to perceive hierarchical structure in equations

**DOI:** 10.1186/s41235-016-0020-9

**Published:** 2016-12-07

**Authors:** Tyler Marghetis, David Landy, Robert L. Goldstone

**Affiliations:** Department of Psychological and Brain Sciences, Indiana University, Bloomington, IN 47405-7007 USA

**Keywords:** Embodied cognition, Neural reuse, Object-based attention, Algebraic reasoning, Rigged Up Perception and Action Systems (RUPAS)

## Abstract

Formal mathematics is a paragon of abstractness. It thus seems natural to assume that the mathematical expert should rely more on symbolic or conceptual processes, and less on perception and action. We argue instead that mathematical proficiency relies on perceptual systems that have been retrained to implement mathematical skills. Specifically, we investigated whether the visual system—in particular, object-based attention—is retrained so that parsing algebraic expressions and evaluating algebraic validity are accomplished by visual processing. Object-based attention occurs when the visual system organizes the world into discrete objects, which then guide the deployment of attention. One classic signature of object-based attention is better perceptual discrimination within, rather than between, visual objects. The current study reports that object-based attention occurs not only for simple shapes but also for symbolic mathematical elements within algebraic expressions—but only among individuals who have mastered the hierarchical syntax of algebra. Moreover, among these individuals, increased object-based attention within algebraic expressions is associated with a better ability to evaluate algebraic validity. These results suggest that, in mastering the rules of algebra, people retrain their visual system to represent and evaluate abstract mathematical structure. We thus argue that algebraic expertise involves the regimentation and reuse of evolutionarily ancient perceptual processes. Our findings implicate the visual system as central to learning and reasoning in mathematics, leading us to favor educational approaches to mathematics and related STEM fields that encourage students to adapt, not abandon, their use of perception.

## Significance

Teaching mathematical skills requires knowing how those skills are actually accomplished by the mathematically proficient. Traditionally, mathematical reasoning was assumed to be divorced from perception and action; pedagogies have thus been devoted to helping students move beyond “superficial” perceptual strategies. There is mounting evidence, however, that mathematical skills actually rely on our perceptual systems, retrained by experience to implement abstract mathematical relations and transformations. The current study investigated one aspect of this perceptual foundation: the use of object-based attention to represent and evaluate hierarchical algebraic relations. Rather than teaching students to do mathematics the way common sense suggests it *should* be done, this basic research on how mathematics is *actually* accomplished could inform the development of educational interventions that treat trained-up perceptual systems as a proper component of mathematical expertise.

## Background

Mathematical practice is undeniably *perceptual*. We read equations, look at geometric diagrams, and inspect graphs. In the canonical mathematical encounter, a mathematician scribbles across a blackboard, writes equations and diagrams, and steps back to inspect their inscriptions. These mathematical inscriptions must be seen to be used.^1^ How should we make sense of all this perception within mathematical activity? The standard account of mathematics—and of mathematical cognition—treats this perceptual labor as decidedly peripheral, even epiphenomenal. On this account, the core feature of mathematics is its abstraction. The competent mathematician, therefore, might use perception to read equations or view diagrams, but should immediately translate that perceptual information into more abstract, perhaps symbolic, internal representations (e.g., Anderson, 2005). Perception and action are merely an interface between the environment and “real” mathematical thinking. The more expert we become, the story goes, the *less* we should rely on superficial visuospatial features (e.g., Kirshner, 1989). Mathematical reasoning should be divorced from the vulgar details of perception and action.

There is certainly something to this account. An algebraic equation has the same meaning whether it is written big or small, with red or black ink. Successful mathematical reasoning requires stripping away superficial, irrelevant details to access the underlying abstract structure. There is a danger, however, of throwing out the perceptual baby with the bathwater of irrelevant detail. There are theoretical and empirical reasons to suppose that perception and action lie at the core of mathematical expertise. Mathematics is too recent a cultural development for humans to have evolved mathematics-specific neural resources. Human mathematical abilities will need to rely on evolutionarily older capacities, recycled for new purposes (Anderson, 2015; Dehaene & Cohen, 2007; Landy, Allen, & Zednik, 2014). Could our perceptual systems be one of those recycled resources?

One context in which the visual system might perform mathematical work is symbolic algebra. Algebraic notation expresses relations that are both abstract and hierarchical, but the notation itself relies heavily on visuospatial features to represent those relations (e.g., Kirshner, 1989; Whitehead, 1911). For instance, algebraic precedence is associated with spatial proximity. While low-precedence operations like addition require a full symbol (p + q), multiplication requires only an abbreviated symbol (*p*•*q*) or no symbol at all (*pq*). If the visual system were sensitive to such regularities, then the hierarchical structure of algebra could be read off directly from an expression’s layout. And, indeed, people are sensitive to these visuospatial norms. When they judge the validity of an algebraic equation, performance is systematically worse if visual grouping or proximity conflicts with operator precedence (e.g., less space around addition than around multiplication), and systematically improved if visuospatial features align with operator precedence (Landy & Goldstone, 2007a; Rivera & Garrigan, 2016). Conversely, when adults write out algebraic expressions, they place terms connected by a higher-precedence operation (e.g., multiplication) closer together than those connected by a lower-precedence operation (e.g., addition; Landy & Goldstone, 2007b). Thus, mathematical notations are designed to tap into pre-existing perceptual biases, grouping related elements according to Gestalt principles (Wagemans et al., 2012), and these design choices have cognitive benefits.

The influence of this formally irrelevant visuospatial information actually *increases* with competence and experience (Braithwaite, Goldstone, van der Maas, & Landy, 2016). This suggests that, over time, people become increasingly sensitive to visual regularities in algebraic notation, perhaps because they are relying more on the notation’s visospatial layout to make algebraic judgments. Mastering a notation’s visuospatial structure allows us to transform symbolic, sequential reasoning of the sort found in mathematics or logic into a series of simpler perceptual tasks (Hutchins, 1995; Rumelhart, Smolensky, McClelland, & Hinton, 1986). As Whitehead (1911, p. 61) observed a century ago, “by the aid of symbolism, we can make transitions in reasoning almost mechanically by the eye, which otherwise would call into play the higher faculties of the brain.”

A more radical possibility is that mathematical experience might actually retrain our perceptual systems so that—in addition to remaining sensitive to the visuospatial structure of the notation itself—they also *impose* perceptual structure onto mathematical representations (Goldstone, Landy, & Son, 2010, Rumelhart et al., 1986). Done right, this would transform symbolic mathematical relations into perceptual structure. One way that our visual system might play this role for algebra—where symbolic, hierarchical relations are critical—is by imposing hierarchical structure on perceived algebraic expressions.

### Object-based attention in vision and reasoning

**Fig. 1:**
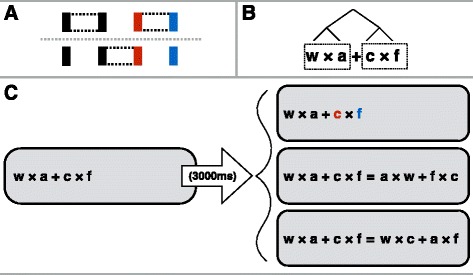
Object-based attention for perceptual and algebraic objects. **a** Visual property verification (e.g., same or different color?) is facilitated when visual elements appear to belong to the same (*top*) rather than different (*bottom*) objects. **b** The syntax of algebra produces hierarchically organized sub-expressions (illustrated by *dotted rectangles*). **c** Trials began with the presentation of an expression (*left*). On Color Verification trials (*top right*), two adjacent variables were changed to either red or blue; participants decided whether the colors were the same or different. If the hierarchical structure of algebra elicits object-based attention, verification should be facilitated within algebraic sub-expressions (e.g., c and f) rather than between them (e.g., c and a). On Algebraic Equivalence trials, a second expression—created by permuting the original expression—appeared to the right, and participants decided whether the expressions were algebraically equivalent. Half the permutations produced expressions that were equivalent—for instance, swapping variables separated by multiplication (*middle right*), which is both commutative and the higher-precedence operation. The other permutations produced expressions that were not equivalent—for instance, swapping variables separated by addition (*bottom right*), the lower-precedence operation

Our perceptual systems constantly construct and impose structure upon the observed environment. The visual system, for instance, imposes structure on sensory input by organizing the visual world into discrete objects (Wagemans et al., 2012). One facet of this structured, hierarchical visual processing is *object-based attention*, in which the visual world is organized into discrete objects, and attending to one part or feature of an object facilitates attention to the rest of the object (Kahneman & Henik, 1981; Kimchi, Yeshurun, & Cohen-Savransky, 2007; Vecera & Farah, 1994). Object-based attention is typically detected in experimental paradigms involving visual property verification. People are better at comparing visual properties (e.g., color) when elements are *within* a single visual object rather than distributed *between* objects (Duncan, 1984; Fig. 1a).

The construction of visual objects does not depend exclusively on sensory cues but is shaped also by experience-dependent expectations. For instance, Zemel, Behrmann, Mozer, and Bavelier (2002) had participants make a comparative judgment about features of objects in a visual scene, but added an occluding object to make it ambiguous whether the features belonged to a single, unusually shaped object or two separate objects. When participants had not observed the unusual shape previously, their responses were consistent with comparing features that belonged unambiguously to separate objects. This suggests that, in line with Gestalt principles, they had interpreted the two parts as belonging to distinct objects. But when participants had previous experience with objects with that unusual shape, they were faster to make the perceptual judgment, as if now they interpreted the two features as belonging to the *same* oddly shaped object. Thus, the visual system constructs objects based not only on sensory cues but also past perceptual experience.

Could mathematical expertise involve adapting object-based attention to perform algebraic reasoning? The rules of algebra—such as the rules governing operator precedence—impose a hierarchical structure that combines simple elements into more complex expressions (Fig. 1b). For instance, when constants and variables are multiplied together, they act as a unified grouping within the larger expression, known as a “term.” Individual terms are then added, subtracted, or combined in other ways to create even more complex expressions, much like words and phrases are combined to form complex sentences. Recognizing this hierarchical structure is critical to algebraic reasoning. When a complex algebraic expression is manipulated, valid manipulations maintain algebraic sub-expressions or act on them in systematic ways; invalid manipulations violate or ignore sub-expressions (Fig. 1c). For instance, given the expression ‘*a • b + x • y*,’ the rules of algebra license swapping the two algebraic sub-expressions, ‘*x • y*’ and ‘*a • b*,’ to get the new expression ‘*x • y + a • b*.’ By contrast, one *cannot* swap the two adjacent variables ‘*b*’ and ‘*x*’ to get ‘*a • x + b • y*.’ This manipulation violates the precedence rules for arithmetic operations. But to detect this violation, it suffices to notice that it breaks apart the two algebraic sub-expressions. Thus, if our visual system were retrained so that—in addition to constructing objects on the basis of sensory cues or experience-based expectations—it also imposed visual objects that were consistent with the requirements of formal mathematics, then attending to these algebraic sub-expressions would be one way for our perceptual systems to accomplish aspects of algebraic reasoning without recourse to abstract, symbolic mental representations. By perceiving algebraic elements that are closer together in a hierarchical structure as a unified, perceptual object, one could transform the conceptual task of verifying algebraic validity into the perceptual task of checking that transformations do not violate algebraic objects.

### Current study

To investigate whether people competent in algebra impose perceptual objects on algebraic expressions, we adapted the property verification paradigm used previously to demonstrate object-based attention (e.g., Baylis & Driver, 1992; Zemel et al., 2002). Participants were first evaluated for mastery of the basic rules that govern the hierarchical structure of algebra (i.e., order of operations). They were then tested for object-based attention within algebraic expressions (e.g., w + a × c + f). On each trial, two adjacent variables changed color, from black to either blue or red, and participants had to determine whether these variables had the same color or different color. If visual objects are constructed based on the expression’s hierarchical structure, then color verification should be facilitated when performed *within* an algebraic sub-expression (i.e., variables separated by multiplication), compared to when performed *between* sub-expressions (i.e., variables separated by addition). Moreover, this within-object advantage should occur only among those participants who have mastered the rules that generate the hierarchical structure of algebra. To investigate whether retraining the visual system modulates algebraic performance, we also tested participants on a purely mathematical task: evaluating the algebraic equivalence of two expressions. If, after participants master the syntax of algebra, their visual system is retrained to play a functional role in algebraic reasoning, then object-based attention for algebraic sub-expressions should improve performance in algebraic reasoning.

## Methods

Following Simmons, Nelson, and Simonsohn (2012), we declare that we report how we determined our sample size, all data exclusions, all manipulations, and all measures. All experimental procedures were approved by the university’s Institutional Review Board (0804000155).

### Participants

Volunteer adults (*N* = 150, *M*_age_ = 20 years; 73 men, 71 women, 6 other gender) participated online in return for partial course credit. Sample size was determined in advance based on a pilot study (*n* = 30), using the same procedure as in the current study, which found object-based attention within algebraic sub-expressions (*p <* *.*05), with evidence for this effect only among participants who had mastered the syntax of algebra (Bayes Factor BF_10_ < 1 for participants who had not mastered algebraic syntax). Based on the effect size of the interaction in this pilot (η_p_^2^ = .02), a sample size of *n* = 135 would have a power of .95 to detect the interaction between Algebraic Term and Syntax Knowledge (Faul, Erdfelder, Lang, & Buchner, 2007).

### Materials

Expressions were displayed on a computer monitor in a monospaced, sans-serif, black font. They consisted of four variables separated by arithmetic operations, either multiplication or addition (Fig. 1c). The symbol for multiplication was created by rotating the addition symbol by 45°. On each trial, variables were represented by a random selection of unique letters from the Roman alphabet—excluding three letters that resemble numerals (*i*, *l*, and *o*) and one that resembled the multiplication symbol (*x*). Expressions had two possible formats: one with multiplication in the center and additions on the outside, and the other with addition in the center and multiplications on the outside. This assured that both arithmetic operations appeared equally in every position within the expressions.

On Algebraic Equivalence trials, initial expressions were joined by a rearranged version, which appeared to the right of an equals sign (Fig. 1c; see Procedure, below). Following Landy and Goldstone (2007a), this second expression was created by applying one of eight possible permutations to the first expression. Half of these permutations produced expressions that were equivalent algebraically to the original; the rest produced expressions that were not equivalent (Fig. 1c).

### Procedure

Participants were first evaluated for their knowledge of the order of precedence for arithmetic operations (“Syntax Knowledge”). Two arithmetic problems with both addition and multiplication (e.g., 4 + 3 × 2 + 1) were followed by four alternatives. One alternative was the correct solution, obtained by performing multiplication before addition (e.g., 11). Other alternatives included the solution obtained if addition were performed before multiplication (e.g., 21) and the solution obtained if operations were completed from left to right (e.g., 15). Participants were considered “Syntax Knowers” if they answered both questions correctly, and “Non-Knowers” otherwise.

Participants then completed the main experimental trials, which involved one of two tasks, assigned randomly on each trial: *Color Verification* or *Algebraic Equivalence*. All trials began with the presentation of an algebraic expression, just left of the display’s midline. The Color Verification task was modeled after the paradigm used by Zemel et al. (2002) to study object-based attention in a purely visual context. On Color Verification trials, 3000 ms after the initial appearance of the algebraic expression, two adjacent variables changed color from black to blue or red (Fig. 1c). This cued participants to determine whether the colored variables were the same color (e.g., both red) or different colors (e.g., one red, one blue). Color Verification trials were used to measure object-based attention. On Algebraic Equivalence trials, the presentation of the initial algebraic expression was followed after 3000 ms by the appearance of a second expression, separated from the first expression by an equals sign (Fig. 1c). This cued participants to determine the algebraic equivalence of the left- and right-side expressions. On both Algebraic Equivalence and Color Verification trials, participants responded by pressing the ‘p’ (same/equivalent) or ‘q’ (different/non-equivalent) keys. Participants were instructed to respond as quickly and accurately as possible; they had up to 10 s to respond, and received immediate feedback after incorrect responses. They completed 336 trials ordered randomly over four blocks, each consisting of 12 Algebraic Equivalence and 72 Color Verification trials.

After completing the main experimental trials, participants reported their age and gender, and responded to a series of questions about their mathematical abilities: mathematics anxiety (from 1 to 10); whether they had completed a college course on finite mathematics (e.g., combinatorics); and their score on the quantitative section of the SAT (which very few participants remembered). No other measures were collected.

### Analysis

For Color Verification trials, Signal Detection Theory (Green & Swets, 1966) was used to analyze perceptual sensitivity while controlling for potential response biases. Pilot results indicated that the effect of object-based attention, in this paradigm, was most pronounced in perceptual sensitivity rather than reaction time.^2^ After removing trials where participants did not respond (<1%), discriminability (*d*’) was calculated for each participant, Algebraic Term (*within* vs. *between* algebraic sub-expressions), and Expression Format (either “*v*_*1*_ + *v*_*2*_ × *v*_*3*_ + *v*_*4*_” or “*v*_*1*_ × *v*_*2*_ + *v*_*3*_ × *v*_*4*_”). Since many participants had perfect discrimination in at least one condition (*n* = 93), 0.25 was added to all cells of the signal detection matrix to correct for infinite estimates of discriminability (Brown & White, 2005). The main results were confirmed by analyses of accuracy (see Appendix).

Analyses were conducted in the R software package (Core Team, 2015). Hierarchical (i.e., mixed-effects) models were fit with the lme4 package (Bates, Maechler, Bolker, & Walker, 2015). All predictors were centered. *P*-values for fixed effects were calculated using Satterthwaite approximations (Kuznetsova, Brockhoff, & Christensen, 2015). Participants were removed for below-chance performance on Algebraic Equivalence trials (*n* = 16) and for poor accuracy (<75%) on Color Verification trials (*n* = 10). Including all participants did not change the pattern or statistical significance of the main results.

## Results

Accuracy was high on both tasks (*M*_color_ = 0.96, *M*_validity_ = 0.81).

**Fig. 2:**
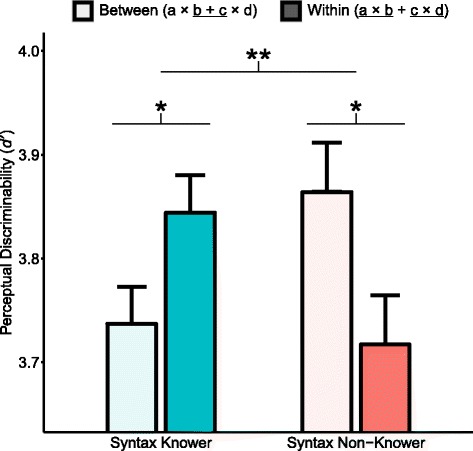
Object-based attention for algebraic sub-expressions. Perceptual discriminability was modulated by whether perceptual comparison occurred *within* an algebraic sub-expression (i.e., variables separated by multiplication) or *between* algebraic sub-expressions (i.e., variables separated by addition). For Syntax Knowers, who exhibited mastery of the rules governing the hierarchical structure of algebra, discriminability was significantly better within algebraic sub-expressions. By contrast, Non-Knowers showed the opposite effect: better discriminability when comparing variables that were separated by lower-precedence addition. Error bars show SEM; *p < 0.05; **p < 0.01

Discriminability (*d*’) on Color Verification trials was analyzed in a mixed ANOVA, with a between-subjects factor of Syntax Knowledge (i.e., *knower* vs. *non-knower*), and within-subjects factors of Expression Format (“v_1_ + v_2_ × v_3_ + v_4_” vs. “v_1_ × v_2_ + v_3_ × v_4_”) and Algebraic Term (*within* vs. *between* algebraic sub-expressions). The only effect that approached significance was the highly significant interaction between Algebraic Term and Syntax Knowledge, F_(1,122)_ = 9.29, *p* = .003 (for all others, *p* > .25. see Fig. 2). This medium-sized effect (η_p_^2^ = .07) was driven by two opposing simple effects. Syntax Knowers (*n* = 78), who know that multiplication has algebraic precedence over addition, had better perceptual discriminability within algebraic terms; that is, when variables that changed color were separated by multiplication rather than by addition, *t*_77_ = 2.1, *p* = .036, Cohen’s *d* = 0.24. In other words, Syntax Knowers showed a significant “within-object advantage” for algebraic sub-expressions, in line with their knowledge of hierarchical structure of algebra. By contrast, Syntax Non-Knowers (*n* = 46) showed the opposite effect, with significantly better discriminability when variables that changed color were separated by addition, *t*_45_ = –2.2, *p* = .033, *d* = –0.33. Thus, perceptual discriminability differed between and within algebraic sub-expressions, modulated by participants’ knowledge of the hierarchical structure of algebra.

These results were confirmed by a linear mixed-effects model, with fixed effects of Algebraic Term, Syntax Knowledge, and their interaction; random effects of Subject and Expression Format; and the maximal converging random effects structure, which had all random intercepts and slopes, uncorrelated (Barr, Levy, Scheepers, & Tily, 2013). Once again, there was a significant interaction between Algebraic Term and Syntax Knowledge (*b* = 0.25, *t* = 2.7, *p* = .007), and this full model was significantly better than a reduced model without the interaction (χ_(1)_ = 4.6, *p* = .03). No other effects were significant (*p* > .85).

### Relations to algebra performance

We next investigated whether object-based attention during Color Verification trials predicted algebraic performance. If mathematically competent undergraduates rely on retrained object-based attention to parse algebraic expressions, then participants who exhibited a greater within-term advantage on Color Verification trials should be better at determining algebraic equivalence. We thus calculated, for each participant, a measure of object-based attention on Color Verification trials, by subtracting mean *d’* on between-term comparisons, from mean *d’* on within-term comparisons. This measure is more positive when discriminability is better for comparisons performed within (vs. between) algebraic term.

First, we verified that Syntax Knowledge facilitated performance on the Algebraic Equivalence task. As expected, participants who had mastered the rules governing order of operations (i.e., Syntax Knowers) were better at evaluating algebraic equivalence (*M* = 83.2% vs. 76.6%), *t*_122_ = –2.5, *p* = .01, Cohen’s *d* = 0.47. To confirm that Syntax Knowledge made a unique contribution to algebra performance, we analyzed trial-by-trial accuracy with a mixed-logit model that included additional fixed effects for available control measures: standardized mathematics anxiety; whether participants had completed college finite mathematics; and, to control for overall engagement, standardized mean accuracy on the Color Verification trials.^3^ The random effects structure was the maximal converging structure motivated by the design, with random effects of Subject and Equation Format, random intercepts, and all random slopes (Barr et al., 2013). Both anxiety (β = –0.22 ± 0.09 SEM, *p* = .016) and performance on Color Verification trials (β = 0.42 ± 0.09 SEM, *p* < .001) were significant predictors of accuracy on Algebraic Equivalence trials. Even after controlling for these factors, however, Syntax Knowledge still predicted algebra performance (*b* = 0.41 ± 0.20 SEM, *p* = .036).

**Fig. 3:**
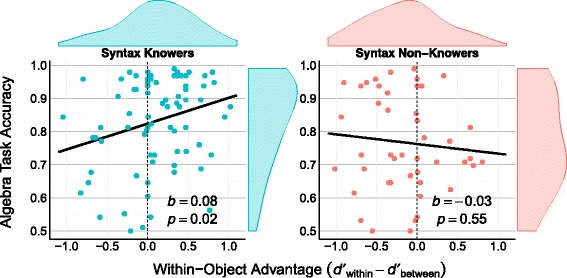
Object-based attention for algebraic sub-expressions predicts algebra performance. Participants’ within-object advantage in discriminability (*horizontal axis*) was used as an index of object-based attention for algebraic sub-expressions. For Syntax Non-Knowers (*right panel*), object-based attention was unrelated to algebra task accuracy (*vertical axis*). For Syntax Knowers (*left panel*), by contrast, algebra performance improved with increasing object-based attention. Dots represent individuals. *Black lines* show lines of best fit that illustrate the relation between individuals’ within-object advantage and their mean accuracy on the algebra task. Density plots show marginal densities for object-based attention (*top*) and accuracy on Algebraic Equivalence trials (*right*)

We next investigated whether object-based attention also facilitated judgments of algebraic equivalence. To the full mixed-logit model of algebra accuracy, we added the measure of participants’ object-based attention, its interaction with Syntax Knowledge, and all associated random slopes. Once again, both anxiety and performance on Color Verification trials predicted algebra performance (both *p* < .01), as did Syntax Knowledge (*p* = .045). The only other significant predictor was the interaction between Syntax Knowledge and object-based attention (*b* = 1.2 ± 0.42 SEM, *p* = .006; Fig. 3). Follow-up subset analyses found that, while Syntax Non-Knowers were overall worse than Knowers at judging algebraic equivalence, their performance was unrelated to their object-based attention (*p* = .17). For the higher-performing Syntax Knowers, by contrast, object-based attention was a highly significant predictor of success in judging algebraic equivalence (*b* = 0.68 ± 0.26 SEM, *p* < .01). Thus, there was evidence that judging algebraic equivalence—a purely mathematical task—was supported by object-based visual attention, but only among those participants who had mastered the basic hierarchical structure of algebra (i.e., Syntax Knowers). Indeed, among Syntax Knowers, our measure of object-based attention accounted for nearly 10% of the variance in participants’ mean accuracy, even after controlling for mathematics education, mathematics anxiety, and overall task engagement.

## Discussion

We investigated the hypothesis that the visual system is retrained to perceive the hierarchical structure of algebraic expressions, reducing high-level algebraic reasoning to basic perceptual processes. As predicted, participants who had mastered the hierarchical structure of algebra exhibited object-based attention for algebraic sub-expressions (i.e., variables around a higher-precedence operation). In addition, the extent of their object-based attention for algebraic sub-expressions predicted their performance on a purely mathematical task, with performance improving as object-based attention increased. This was not the case for participants who had not yet mastered the hierarchical structure of algebra; they did not exhibit object-based attention for algebraic structure, and their algebraic performance was unrelated to their perceptual processing. Taken together, these results are consistent with the hypothesis that mathematical expertise involves, at least in part, recycling processes in the visual system to create structured groups that honor the hierarchical structure of algebra.

Why, for some participants, was perceptual discriminability actually better between algebraic sub-expressions than within? Most of these participants were Syntax Non-Knowers. Some of these individuals may have the order of precedence exactly wrong, solving addition first—perhaps because it is easier—and only afterwards moving to multiplication. Past studies have found that a full third of college students struggle to apply the correct order of operations (Pappanastos, Hall, & Honan, 2002; see also Glidden, 2008). Perhaps more likely, the addition symbol may attract attention for purely visual reasons (e.g., it consists of lines that are vertical and horizontal, rather than slanted) or because it is more familiar, comfortable, and comprehensible, particularly for lower-performing individuals. Indeed, extensive early experience with addition may train the visual system to perceive sums as wholes, an early bias that must be overridden by later algebraic training.

A between-object advantage, however, was found even among some Syntax Knowers—including a few who performed quite well on the Algebraic Equivalence task. Some of this is presumably just noise; no behavioral index of object-based attention is going to be a perfect measure of perceptual processing. But this is also a good reminder that there are multiple routes to mathematical success. It is unlikely that every competent reasoner is going to rely on the same visuospatial perceptual strategy; some may even rely entirely on rote, explicit, linguistically encoded knowledge of the order of operations (e.g., recalling the abbreviation PEDMAS: Parentheses, then Exponents, then Division and Multiplication, then Addition and Subtraction). Object-based attention for algebraic structure, therefore, may take time to develop, emerging only after mastering algebraic syntax. For some, perceptual processes may always be overshadowed by complementary strategies.

Previous work has demonstrated object-based attention for concrete objects inferred from sensory cues (Duncan, 1984) and expectations that reflect past perceptual experience (Zemel et al., 2002). The current study extended this phenomenon to objects established on the basis of abstract relations and conceptual knowledge. In some ways, this is reminiscent of the holistic perception of written words (Ehri, 2005). Skillful readers retrain their visual system so they see written words as wholes, not collections of individual letters. Holistic word perception, however, still depends primarily on a sensory cue—the space between words—or past exposure to that particular word-form. This is sometimes true for algebraic notation, too, where algebraic precedence is associated with spatial proximity. Often, however, the hierarchical structure of an expression is not readily apparent from visual inspection alone. In the current study, for instance, addition and multiplication were spaced equally, minimizing any sensory cues indicating which variables belong together. Furthermore, during reading, only specific combinations of letters form legitimate words. In algebra, by contrast, new variables can be combined productively to create novel sub-expressions; indeed, in the current study, letters were chosen randomly from the alphabet, generating combinations that participants may have never before encountered. Despite this productive novelty, algebraic sub-expressions were perceived as unified visual objects. These visual objects could only have been constructed on the basis of the formal rules governing algebraic syntax. Basic perception was reshaped by high-level conceptual knowledge.

### The nature of mathematical expertise

The current results suggest that relying on visual processing might be a boon, not a barrier, to mathematical reasoning. This might come as a surprise. Confronted with evidence of students’ reliance on misleading, superficial visual strategies in algebra, some have argued that mathematical training should avoid and even suppress perceptual strategies (e.g., Kirshner, 1989; Kirshner & Awtry, 2004). For example, when asked to solve 4 + 4/2 + 2, some students might be led to answer “2,” incorrectly, because of the superficially tempting, perceptually strong 4 + 4 and 2 + 2 groups. Indeed, we sometimes found evidence for perceptual grouping around addition, rather than multiplication, particularly among participants who had yet to master the hierarchical syntax of algebra. But the fact that novices use perceptual strategies to arrive at incorrect answers does not imply that experts abandon such strategies entirely. Instead, experts may refine those perceptual strategies so that they become reliable, robust, and rapid routes to *correct* solutions (Goldstone et al., 2010; cf., Hutchins, 1995, and Rumelhart et al., 1986). In line with this, participants who had mastered the hierarchical syntax of algebra also exhibited object-based attention for algebraic sub-expressions. Mathematical expertise, therefore, might be better thought of as the *skillful* deployment of perception.

Thus, the mathematical expert is made more expert, on the one hand, by mastering clever notations in which conceptual relations are presented perceptually and, on the other, by retraining their visual system to perform some aspects of algebraic reasoning. Both this perspective on mathematical practice and its resistance have a long heritage. To quote Whitehead (1911, p. 61) yet again: “It is a profoundly erroneous truism, repeated by all copy-books and by eminent people when they are making speeches, that we should cultivate the habit of thinking what we are doing. The precise opposite is the case. Civilization advances by extending the number of important operations which we can perform without thinking about them.” The resistance continues to this day. *New Mathematics* was a relatively recent, and particularly controversial, movement in education that attempted to foreground the “important operations” of mathematics, at the expense of procedural mastery (Adler, 1972). But one implication of our perspective is that mathematical training might be better spent encouraging students to adapt—not abandon—their perceptual grouping processes. Instead of minimizing students’ reliance on perceptual strategies (Kirshner, 1989; Kirshner & Awtry, 2004), education should aim to refine students’ use of perception and action, so that they rig up their perception and action systems like mathematical experts. This could take the form of explicit instruction on how the visuospatial layout of algebraic equations contains hints to the hierarchical relations that they represent. Additionally, future curricula or tools could intervene in targeted ways on the embodied routines that contribute to mathematical expertise, taking advantage of decades of research on perceptual and motor learning (Ottmar & Landy, in press).

Regardless of what we do as teachers, children pick up on the perceptual regularities of their environments, implicitly developing perceptual associations and routines. These can become obstacles, such as when children interpret the visual form of the equals sign as a cue to calculate, hindering learning in early algebra (McNeil, 2008). But they can also offer long-term benefits, such as the perceptual strategy documented in the current study. We imagine a future where computer-based tools will systematically manipulate the visual and interactive features of mathematical representations so that children pick up on the perceptual regularities that help, rather than hurt (e.g., Weitnauer, Landy, & Ottmar, 2016).

Of course, perception alone is insufficient to account for all of mathematical reasoning. However, we suspect it is a critical part of the larger, distributed system that accomplishes mathematics, a system in which resources within the skull are brought into coordination with resources outside (e.g., gestures, inscriptions), skillfully soft-assembled to respond to the situated demands of the task (Clark, 2008). These sundry resources are often “embodied,” from neural circuits that evolved for perceiving and acting, to the fleshy hands that do the literal “manual labor” of mathematics (Marghetis, Edwards, & Núñez, 2014). For example, brain circuits that evolved for perceiving motion or shifting attention are redeployed to support mathematical skills like symbolic arithmetic, where attention is shifted along a simulated number-line (Knops, Thirion, Hubbard, Michel, & Dehaene, 2009; Marghetis, Núñez, & Bergen, 2014; McCrink, Dehaene, & Dehaene-Lambertz, 2007), or solving equations, where terms are imagined to move across the equals sign (Goldstone et al., 2010). Our bodies, too, are disciplined by mathematical training. When a mathematical expression is examined, eye movements respect the expression’s hierarchical structure, starting with the highest-precedence operation and moving sequentially to gradually lower-precedence operations (Landy, Jones, & Goldstone, 2008; Schneider, Maruyama, Dehaene, & Sigman, 2012). And while gestures can shape children’s early mathematical knowledge (Goldin-Meadow, Cook, & Mitchell, 2009), even experts gesture spontaneously to express their mathematical understanding (Marghetis & Núñez, 2013). A complete understanding of mathematical cognition requires that we study mathematics as it is actually accomplished, as an embodied practice: eyes darting across the blackboard, hands scribbling away.

### The widespread role of regimented perception

While the current study has focused on retraining our perceptual apparatus to perform *algebraic* reasoning, mathematics is full of other practices that also likely depend on the regimentation of perception. Visual proofs in Euclidean geometry are unreliable when treated naively as exact depictions, but the expert geometer learns to ignore those diagrammatic features that could lead to invalid conclusions (e.g., exact length) while perceiving those features that can make valid contributions to a proof (e.g., containment; Manders, 2008). And this is not restricted to high school mathematics. Category Theory, a branch of modern mathematics, relies on a proof technique known as “diagram chasing” that relies entirely on the creation and interpretation of diagrams in which spatial locations indicate mathematical relations. Indeed, visuospatial ability is significantly greater among professional mathematicians compared to non-mathematicians, and it completely mediates the relation between basic numerical abilities and the attainment of advanced mathematical expertise (Sella, Sader, Lolliot, & Cohen Kadosh, 2016). Thus, while algebra has been our case study, we propose that mathematics more generally depends for its accomplishment on the cultural regimentation of our perceptual apparatus.

And this may be an even more general phenomenon, with regimented perception playing a role in the reproduction of many, if not most, sociocultural systems. Biases in face perception, for instance, may contribute to the reproduction of structural racism: implicit racial biases, which shape the perception of facial emotions (Hugenberg & Bodenhausen, 2003), can influence split-second decisions by law enforcement about whether or not to shoot a suspect (Correll, Park, Judd, & Wittenbrink, 2007), thus reproducing structural inequalities in safety and policing. Marx even argued that a similar process of regimented perception contributes to the reproduction of capitalist society as a whole, such that we learn to *see* the world in terms of objects to be owned (Marx, 2012). Thus, the cultivation of highly disciplined ways of seeing and acting may be a critical mechanism by which we reproduce immense sociocultural systems (Bourdieu, 1977), from structural inequality to the inferential structure of mathematics.

## Conclusions

Let us return to the puzzle with which we began: why is mathematical practice so thoroughly perceptual? Our answer is that the mathematical expert need not abandon a reliance on perception. Perception is not an obstacle to abstraction. On the contrary, culturally regimented perception is the engine of expert mathematical reasoning. In particular, high-level algebraic reasoning is accomplished by basic perceptual processes that are adapted to reflect abstract conceptual knowledge. Difficult conceptual tasks are thus transformed into robust perceptual ones. This exemplifies a strategy that recurs throughout human cognition: perception and action are rigged up so that “the senses have therefore become directly in their practice theoreticians” (Marx, 2012, p. 107).

## Appendix

### Confirmatory analyses of accuracy

In addition to analyzing discriminability, which controls for any response bias, we also further confirmed all main findings with analyses of accuracy. Accuracy on Property Verification trials was analyzed with a mixed-logit model, with fixed effects of Algebraic Object, Syntax Knowledge, and their interaction; random effects of Subject, Equation Format, and Comparison Location (first, second, or third location with the expression); and the maximal converging random effects structure, which had all random intercepts and slopes, uncorrelated. The only significant effect was once again the interaction between Algebraic Object and Syntax Knowledge (*b* = 0.51, *z* = 2.1, *p* = .03). Follow-up subset analyses revealed that Syntax Knowers were significantly more likely to be correct if they were judging colors within a *multiplication* object (*M* = 97% vs. 96%; *b* = 0.19, *z* = 2.16, *p* = .03). Syntax Non-Knowers, by contrast, were marginally more likely to be correct within an *addition* object (*M* = 97% vs. 96%; *b* = –0.30, *z* = –1.89, *p* = .06).

## Footnotes

1 Or, for blind mathematicians, they must be *touched*.

2 Exploratory analyses of the current study, suggested by reviewers, hints at one possible explanation for this: Syntax Knowers’ reaction times were less variable, *p* = .01. Thus, any evidence of object-based attention in reaction times would have been compressed in exactly that subset of participants who were predicted to show the largest effect. There was no such compression in discriminability.

3 SAT scores were not included because so few participants (<25%) reported their score.
